# Widefield Two-Photon Excitation without Scanning: Live Cell Microscopy with High Time Resolution and Low Photo-Bleaching

**DOI:** 10.1371/journal.pone.0147115

**Published:** 2016-01-29

**Authors:** Rumelo Amor, Alison McDonald, Johanna Trägårdh, Gillian Robb, Louise Wilson, Nor Zaihana Abdul Rahman, John Dempster, William Bradshaw Amos, Trevor J. Bushell, Gail McConnell

**Affiliations:** 1 Centre for Biophotonics, Strathclyde Institute of Pharmacy and Biomedical Sciences, University of Strathclyde, Glasgow, United Kingdom; 2 Strathclyde Institute of Pharmacy and Biomedical Sciences, University of Strathclyde, Glasgow, United Kingdom; 3 MRC Laboratory of Molecular Biology, Cambridge Biomedical Campus, Cambridge, United Kingdom; Pennsylvania State Hershey College of Medicine, UNITED STATES

## Abstract

We demonstrate fluorescence imaging by two-photon excitation without scanning in biological specimens as previously described by Hwang and co-workers, but with an increased field size and with framing rates of up to 100 Hz. During recordings of synaptically-driven Ca^2+^ events in primary rat hippocampal neurone cultures loaded with the fluorescent Ca^2+^ indicator Fluo-4 AM, we have observed greatly reduced photo-bleaching in comparison with single-photon excitation. This method, which requires no costly additions to the microscope, promises to be useful for work where high time-resolution is required.

## Introduction

Widefield single-photon microscopy is still extensively used for live cell Ca^2+^ imaging using fluorescent reporter molecules. However, as well as problems with loading, compartmentalisation and leaking of fluorescent Ca^2+^ indicators, one of the main limitations of their use in single-photon microscopy is photo-bleaching, which can severely limit the number of experiments possible with a given specimen and restrict the useful duration of imaging experiments [1]. Here we demonstrate a new method of live cell Ca^2+^ imaging using widefield two-photon excitation without scanning, which has the advantage of greatly reduced photo-bleaching in comparison with single-photon excitation.

Immediately after its introduction by Denk, Strickler and Webb in 1990 [2], two-photon excitation of fluorescence was adopted in many fields of biomedicine. Its chief advantages are its ability to penetrate more deeply into tissues than single-photon excitation, the creation of optical sections (by the combination of an excitation proportional to the square of the intensity with the conical beam geometry) and the more efficient utilization of scattered emission than is possible in a confocal microscope. The chief drawbacks were the slow scanning speed, which restricted the original instruments to a rate of approximately one image per second, often unusable for electrophysiology, where millisecond time resolution may be needed, and the high rate of photo-bleaching if the laser intensity was increased [3]. To overcome the slowness, faster scanning mirrors have been used [4], and parallelism has been achieved in two-photon imaging by the use of slit scanning [5] or the scanning of multiple foci, preferably uncorrelated in time [6–8]. More recently, Konnerth and co-workers developed a powerful method they refer to as low-power temporal oversampling (LOTOS) to achieve image acquisition rates of 1,000 Hz (28 *μ*m × 9 *μ*m frame size) using an acoustic-optic deflector (AOD) for the fast x-scan [9, 10], and 200 Hz (27–42 *μ*m field of view) using a conventional 12-kHz resonant mirror instead of the AOD [11]. Two-photon light-sheet microscopy has also been used to increase the rate of imaging [12], but because scattering and absorption in the specimen causes inhomogeneous illumination, this method is best suited to highly transparent tissue volumes. A simple and elegant approach of illumination with a large stationary spot of light from a mode-locked femtosecond-pulsed laser in a conventional microscope and imaging of the emission from structures within the spot, though not favoured by Fittinghoff *et al* who were apparently the first to try it [6], was shown by Hwang *et al* to provide images of good quality [13]. We show how this unexpected result can be explained partly by the increased exposure time of the widefield method and partly by the capture of signal from additional fluorophores above and below the plane of focus in the widefield case.

The work of Hwang *et al* on several medically-important specimens seems to have only a limited effect on normal practice, possibly because the illuminated spot was no greater than 60 *μ*m in diameter and the loss of optical sectioning has been regarded as serious. We have repeated and extended this approach, increasing the field size to a diameter of 90 *μ*m and concentrating on fast transients in living neurones, a specimen where the potentially unlimited time resolution of the stationary beam can be evaluated.

In this work, we have used modest average powers from an 80 MHz repetition rate femtosecond-pulsed Ti:Sapphire laser and have collected images using a sensitive sCMOS camera at image acquisition rates of up to 100 Hz. All components of our simple microscope setup are commercially available. We have explored the image quality, phototoxicity and bleach rate at different framing rates, making extended video recordings of synaptically-driven Ca^2+^ events in live neurones loaded with Fluo-4 AM, which we have chosen because they show both slow and fast activity. Our chief new finding is that photo-bleaching is greatly reduced in the widefield two-photon regime relative to that seen with single-photon excitation. We have shown that this unexpected advantage relative to normal practice is particularly marked at high framing rates.

## Materials and Methods

### Excitation and detection of two-photon fluorescence

Awareness of the high peak intensities required for two-photon excitation has probably deterred experimentation on widefield two-photon microscopy. Here we compare excitation and detection parameters in the widefield two-photon microscope with that in a standard point-scanning two-photon microscope.

For both point-scanning and widefield two-photon excitation, we used a wavelength of *λ* = 840 nm, repetition frequency Δ*ν* = 80 MHz and pulse duration *τ* = 140 fs and either a 40x/1.30 NA or a 60x/1.35 NA oil immersion objective for experiments with fibres stained with acridine orange. We assume that pulse stretching is negligible, and is similar in both the laser scanning and widefield microscopes.

Using a handheld laser power meter (Ophir Photonics), we measured a time-averaged power of *P*_*av*_ = 1.8 mW at the specimen plane in the point-scanning microscope (Leica SP5). With the pulse duration *τ* and pulse repetition frequency Δ*ν*, we obtain a peak power of *P*_*peak*_ = 160.71 W from

$$\begin{array}{c} P_{peak}=\frac{P_{av}}{\tau \Delta \nu }. \end{array}$$ (1)

The beam waist radius (*w*_0_) of the excitation laser at the specimen plane is given by [14]

$$\begin{array}{c} w_{0}=\frac{\lambda }{\pi \theta }, \end{array}$$ (2)

where *θ* is the half-angle of the beam divergence given by the numerical aperture

$$\begin{array}{c} NA=nsin\theta . \end{array}$$ (3)

For the 40x/1.30 NA oil immersion lens (*n* = 1.52), *θ* = 1.03 rad and thus at a wavelength of 840 nm, the beam waist radius is *w*_0_ = 260 nm.

The peak intensity in the excitation spot is given by

$$\begin{array}{c} I_{peak}=\frac{P_{peak}}{\pi {w_{0}}^{2}}, \end{array}$$ (4)

and from the calculated values of *P*_*peak*_ and *w*_0_, we obtain a peak intensity of *I*_*peak*_ = 7.57 × 10^14^ W/m^2^ for point-scanning.

In the widefield two-photon microscope, we used a time-averaged power of *P*_*av*_ = 500 mW at the specimen plane, which gives a peak power of *P*_*peak*_ = 44,643 W from Eq (1).

Instead of focusing to a diffraction limited spot using the 60x/1.35 NA lens by overfilling the back aperture, we instead focus to a small spot near the back aperture to obtain a weakly focusing spot at the specimen plane with a radius of *w*_0_ = 45 *μ*m. Using these values of *P*_*peak*_ and *w*_0_ in Eq (4) above, the peak intensity in the widefield two-photon microscope is therefore *I*_*peak*_ = 7.02 × 10^12^ W/m^2^. We note that this peak intensity is two orders of magnitude lower than the peak intensity for a point-scanning two-photon microscope.

The substantially lower peak excitation intensity in the widefield two-photon microscope is compensated in part by negating the need to scan the beam. By continuously irradiating across the whole field, we can use a much longer integration time for collection of fluorescence.

In the Leica SP5 point-scanning two-photon microscope, we used a scan speed of 400 Hz, which, for an image that is 320 × 270 pixels (the same image size we use in the widefield setup), gives a scan period of 7.8125 *μ*s, from $\frac{1\;s}{400\;lines}\times \frac{1\;line}{320\;pixels}$. Since only 30% of the scan period is used for collecting the fluorescence emission, this yields a pixel dwell time of T = 2.34 *μ*s. Since the fluorescence intensity in two-photon excitation depends on the square of the peak intensity of the excitation light, the fluorescence in the point-scanning microscope is therefore proportional to $I_{peak}^{2}$ × T = 1.34 × 10^24^ W^2^⋅s^2^/m^4^.

In the widefield two-photon microscope, the specimen is continuously irradiated with the femtosecond-pulsed laser, but in comparison with the point-scanning two-photon microscope we can consider the dwell time to be equivalent to the frame rate of the camera. For images taken at 100 Hz (i.e. a T = 10 ms exposure time), the fluorescence intensity is proportional to $I_{peak}^{2}$ × T = 4.93 × 10^23^ W^2^⋅s^2^/m^4^. This is 2.7× smaller than in point-scanning. This 2.7× factor may well be accounted for in the widefield two-photon microscope in the following way: The geometry of the widefield two-photon excitation beam is such that instead of being focused to a diffraction-limited spot, it is a weakly focusing spot at the specimen plane, with a beam waist radius of 45 *μ*m. This means that there is a relatively uniform level of two-photon excitation at all axial levels in the excitation beam, and this uniformity in two-photon excitation, extending to tens of micrometres axially (in contrast to being only about half a micrometre in point-scanning), coupled with the additional fluorescence signal from all axial levels within the photometric volume (the conical volume defined by the pixel size and the numerical aperture of the objective), accounts for the usable levels of detected fluorescence even though the excitation intensity has decreased drastically. Moreover, the quantum efficiency of an sCMOS camera (60% for the Zyla camera) is higher than that of a photomultiplier tube used in a point-scanning two-photon microscope (∼30%), therefore offering the possibility of high detection sensitivity.

### Experimental setup

**Fig 1:**
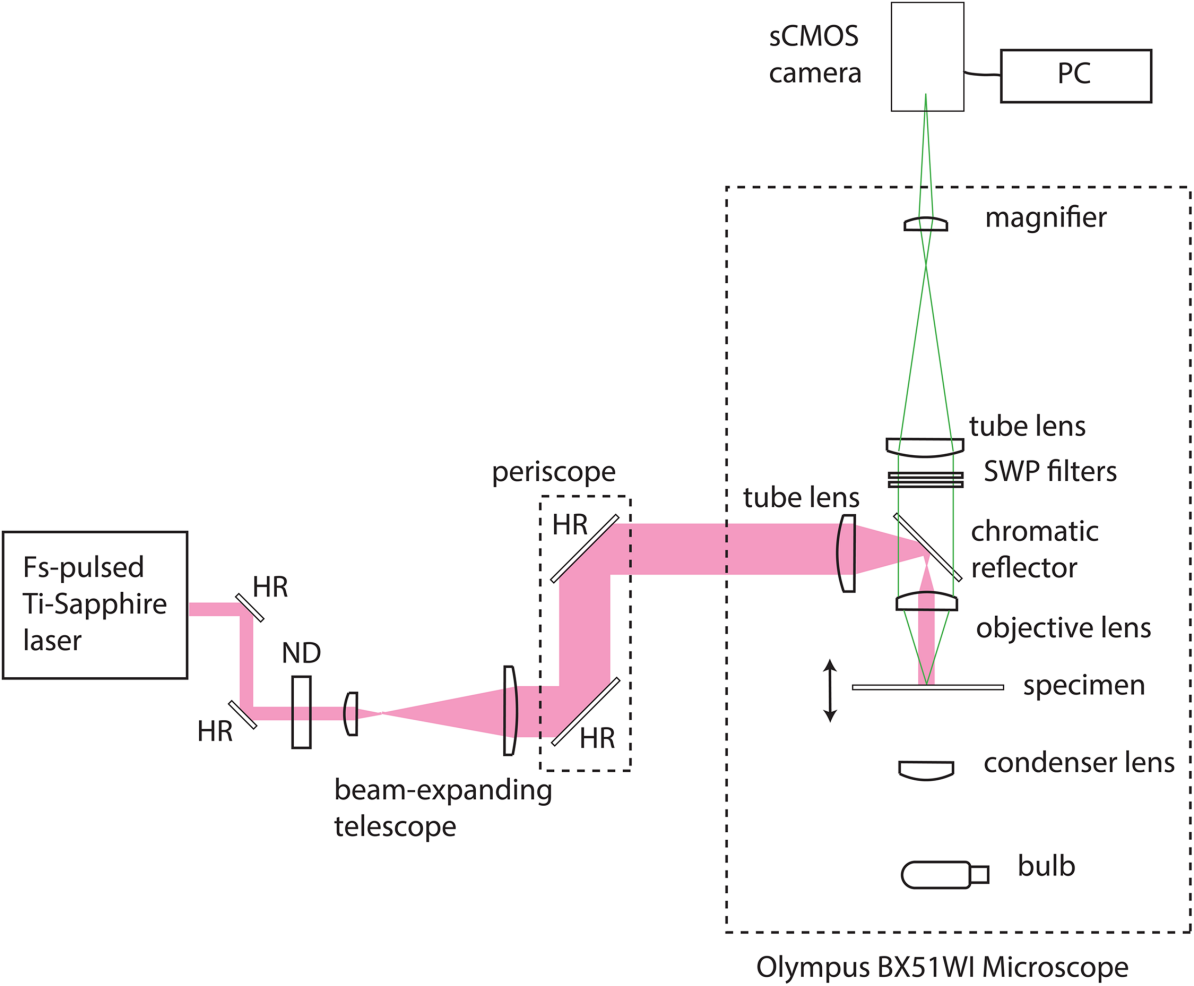
Widefield two-photon microscope and image capture system. An upright microscope was modified for two-photon excitation by changing the excitation, emission and dichroic filters. A cooled sCMOS camera was used to detect fluorescence from the specimen. A femtosecond-pulsed Ti:Sapphire laser was used as the excitation source. The weakly divergent output from the Ti:Sapphire laser was coupled into the microscope and focused to provide a beam waist close to the back aperture of the objective lens. This produced a wide and weakly-focused beam in the specimen plane which did not contribute significantly to the optical sectioning power. HR = highly reflecting mirror, ND = neutral density filter, SWP = short-wave pass filter.

Our simple widefield two-photon microscope was configured as shown in Fig 1. A commercial upright epi-fluorescence microscope (BX51WI, Olympus) was modified for two-photon excitation. A chromatic reflector (FF670-SDi01, Semrock) reflecting wavelengths longer than 670 nm and transmitting shorter wavelengths was used to direct excitation towards the specimen and to transmit fluorescence. A cooled sCMOS camera (Zyla 5.5, Andor) controlled by a PC running the freely available software WinFluor [15] was used to detect fluorescence from the specimen. For blocking of the laser source from the camera and to ensure that only the fluorescence signal contributed to the image, we used 680 nm and 694 nm short-wave pass filters (FF01-680/SP and FF01-694/SP, Semrock) in the collection path. A commercially available wavelength-tunable femtosecond-pulsed Ti:Sapphire laser (Chameleon Ultra II, Coherent) was used as the excitation source. This laser delivered a maximum time-averaged power of 2.3 W at a repetition rate of 80 MHz with a pulse duration of 140 fs. The weakly divergent output from the Ti:Sapphire laser was steered using a pair of highly reflecting mirrors (BB1–E03, Thorlabs) and then attenuated using a variable neutral density filter (NDC-25C-4, Thorlabs). We used a plano-convex lens pair of focal lengths 35 mm and 100 mm (LA1027-B and LA1509-B, Thorlabs) separated by slightly greater than the sum of their focal lengths, to produce a convergent beam with an initial diameter of 22 mm. The beam height was changed using a two-mirror periscope (BB1–E03, Thorlabs) to permit coupling of the laser into the upright microscope. At height, the beam was focused using a single plano-convex lens of focal length f = +75 mm (LA1608-B, Thorlabs), with the beam waist close to the back aperture of the objective lens. This produced a wide and weakly-focused beam in the specimen plane which did not contribute significantly to the optical sectioning power. The illuminated area had a diameter of 90 *μ*m and a 1.6× magnifier was used to fill the camera sensor. A 60x/1.35 NA oil immersion lens was used to image the fluorescent fibres and a 60x/0.9 NA water dipping lens was used to obtain all live cell imaging data.

For single-photon widefield imaging experiments, the short-wave pass filters were removed and the long-wavelength chromatic reflector was replaced with a filter cube (XF22, Omega Optical, Inc.) that comprised an excitation filter with a peak wavelength of 485 ± 22 nm, a chromatic reflector with a 505 nm long-wave pass filter and an emission filter with a peak wavelength of 530 ± 30 nm. The 75 mm plano-convex tube lens was removed, and the mercury arc lamp excitation source (U-ULS100HG, Olympus) and coupling optics originally supplied with the microscope were reattached. At a wavelength of 485 nm, the maximum time-averaged power at the specimen plane exiting the 60x/0.9 NA water dipping objective lens was 240 *μ*W. The same camera was used with the same PC and controlling software as with widefield two-photon excitation.

### Photo-bleaching experiments using fluorescent fibres

We performed photo-bleaching experiments on lens tissue fibres stained with acridine orange, acquiring recordings lasting 600 seconds at image acquisition rates of 1 Hz, 10 Hz and 100 Hz using both widefield single-photon and two-photon excitation. Lens tissue (Whatman, Sigma-Aldrich, UK) cut into 10 mm × 10 mm squares were stained with 10 *μ*L of 120 *μ*M acridine orange in phosphate buffered saline (PBS, D8537, Sigma-Aldrich, UK) and mounted in 10 *μ*L of 90% glycerol / 10% PBS. A coverslip was lowered gently over the mounting medium at an angle, allowing the liquid to spread out and the edges sealed with nail varnish. For single-photon excitation we used the mercury arc lamp (U-ULS100HG, Olympus) and the XF22 filter cube (Omega Optical, Inc.). For two-photon excitation of the acridine orange, we used the 840 nm output of the Ti:Sapphire laser and the FF670 chromatic reflector (Semrock) to direct the long-wavelength excitation light towards the specimen and transmit light of wavelengths shorter than 670 nm, and 680 nm and 694 nm short-wave pass filters (FF01–680/SP and FF01–694/SP, Semrock) to detect the fluorescence. In our widefield experiments, we had to increase the average power of the excitation light reaching the specimen as the framing rate was increased. With single photon excitation the power was 0.035 *μ*W at 1 Hz, 0.3 *μ*W at 10 Hz and 3 *μ*W at 100 Hz. The corresponding powers for two-photon excitation were 25 mW, 150 mW and 500mW. Five specimens were used for each imaging condition, with two recordings made for each specimen and the fluorescence intensity measured over three regions of interest (ROIs) within a fibre using WinFluor. The fluorescence measurements were exported as text from WinFluor, opened in MS Excel and MATLAB and normalised to the fluorescence intensity at the start of each recording and averaged over all recordings (30 for each imaging condition).

We also performed the same experiment on lens tissue fibres stained with 10 *μ*M primuline [16] in tap water and mounted in 90% glycerol / 10% PBS. With single photon excitation, the power was 12 *μ*W at 1 Hz, 260 *μ*W at 10 Hz and 1.04 mW at 100 Hz. The corresponding powers for two-photon excitation were 33 mW, 110 mW and 180 mW. Three specimens were used for each imaging condition, with two recordings made for each specimen and the fluorescence intensity measured over three ROIs within a fibre using WinFluor. The fluorescence measurements were exported as text from WinFluor, opened in MS Excel and normalised to the fluorescence intensity at the start of each recording and averaged over all recordings (18 for each imaging condition).

### Photo-bleaching experiments using fixed cells

We carried out further photo-bleaching experiments on NIH 3T3 cells fixed and stained with FITC Phalloidin. We imaged over a period of 600 s for each acquisition rate for both widefield single-photon and widefield two-photon excitation. The 3T3 cells were maintained in a humidified incubator at 37°C / 5% CO_2_ and were grown in Dulbecco’s Modified Eagle’s Medium (DMEM) supplemented with 10% fetal bovine serum and 5% penicillin streptomycin. Cells were passaged via standard protocols, and plated out onto glass coverslips prior to imaging experiments. The cells were fixed and stained after an 80% confluent monolayer was reached. The medium was then removed and the monolayer washed three times in PBS before fixation in 4% paraformaldehyde for 10 minutes. The coverslips were washed again in PBS before incubating in 0.1% Triton X-100 in PBS for 5 minutes for permeabilisation. The cells were washed again after permeabilisation and then stained by incubation for 20 minutes in FITC Phalloidin (F432, Life Technologies) diluted in PBS to a concentration of 6 units per coverslip. The cells were washed in PBS before being mounted in 90% glycerol / 10% PBS. The light sources and filters used were the same as those used for the photo-bleaching studies on fluorescent fibres. With single-photon excitation, the power was 6.5 *μ*W at 1 Hz, 150 *μ*W at 10 Hz and 500 *μ*W at 100 Hz. The corresponding powers for two-photon excitation were 550 mW, 800 mW and 1000 mW. Three specimens were used for each imaging condition, with two recordings made for each specimen and the fluorescence intensity measured over six ROIs across the field of view. The fluorescence measurements were exported and normalised as described above.

### Imaging primary rat hippocampal cultures

We also performed widefield two-photon microscopy of primary rat hippocampal cultures to demonstrate the capability of rapid imaging. Primary rat hippocampal cultures were prepared as described previously [17]. Briefly, Sprague-Dawley rat pups (1–2 days old) were obtained from in-house colonies maintained in the Biological Procedures Unit at the University of Strathclyde. The Biological Procedures Unit has the appropriate permits and is required to report animal usage, and animal care and followed standard protocols of basic housing and breeding up to sacrifice. All animal care and experimental procedures were in accordance with the guidelines of the United Kingdom Home Office under the agreement and authority of the United Kingdom Animals (Scientific Procedures) Act 1986. The pups were killed by cervical dislocation and decapitation following Schedule 1 procedures of the United Kingdom Animals (Scientific Procedures) Act 1986, and the brain was removed. The hippocampi were then dissected out, incubated in a papain solution (1.5 mg/ml, Sigma-Aldrich) at 37°C for 20 minutes. The hippocampi were then washed in solution containing bovine serum albumin (10 mg/ml), dissociated by trituration and plated onto coverslips previously coated with poly-L-lysine (0.1 mg/ml) at a final density of 3 × 10^5^ cells/ml. Cultures were incubated in Neurobasal-A Medium (Invitrogen) supplemented with 2% (v/v) B-27 (Invitrogen) and 2 mM L-glutamine and maintained in a humidified atmosphere at 37°C/5% CO_2_. After 5 days *in vitro* (DIV), cytosine-D-arabinofuranoside (10 *μ*M) was added to inhibit glial cell proliferation. Cells were used experimentally from 11–14 DIV.

For imaging, hippocampal cultures were washed twice with a HEPES-buffered saline (HBS) containing (in mM): NaCl 140, KCl 5, MgCl_2_ 2, HEPES 10, D-glucose 10 and CaCl_2_ 2, pH 7.4, and transferred to HBS containing Fluo-4 AM (10 *μ*M, 45–60 min, room temperature). Once loaded, cells were washed with HBS and images were obtained from the cell bodies of neurones with constant irradiation and with frame rates of up to 100 Hz. Pixel binning was used to increase the signal to noise ratio of the images: four pixel binning was used at frame rates of 1–50 Hz, and eight pixel binning was used for camera frame rates of 100 Hz. Experiments were performed on cultures at room temperature. Cells were identified as neuronal cell bodies based on bright-field morphology, and ROIs were chosen in cell bodies using Winfluor. For each dataset analysed, an ROI outside of the cell was also chosen as the background. The signal intensity count of the background ROI was subtracted from the signal intensity count of all cell ROIs in each dataset and these corrected ROI time course values were exported in text format using Winfluor. These data were opened in MS Excel and MATLAB, and were normalised to the signal intensity values at time t = 0 s. The sample sizes for the different imaging speeds were: for single-photon excitation, n = 23 at 1 Hz, n = 21 at 10 Hz and n = 98 at 100 Hz, and for two-photon excitation, n = 30 at 1 Hz, n = 35 at 10 Hz and n = 24 at 100 Hz, where n is the number of cells analysed with data acquired from at least three separate cultures.

We compared the measured fluorescence intensity over time in the live cell neuronal preparations using both single-photon and two-photon widefield excitation to investigate photo-bleaching at different image acquisition rates. For these measurements, we used time-averaged powers of ∼50 *μ*W for single-photon and ∼100 mW for two-photon excitation at 1 Hz, ∼400 *μ*W and ∼170 mW at 10 Hz, and ∼2.5 mW and 360 mW at 100 Hz. These excitation powers were chosen to facilitate the best comparison of single-photon and two-photon excitation and varied by less than 20% for each image acquisition rate. Similar initial fluorescence signal intensity counts were obtained by adjusting the excitation radiation in one region of the specimen then moving to an adjacent region for recording. It is perhaps helpful to note here that this setup procedure was possible by eye, since the two-photon excitation of fluorescence could be observed using the binocular viewer of the microscope. To our knowledge, this is the first observation of widefield two-photon excited fluorescence using the binocular viewer of the microscope when an 80 MHz repetition rate (non-amplified) Ti:Sapphire laser is applied.

To determine whether the transient changes in fluorescence signal intensity over time were caused by synaptically-driven events, NBQX (20 *μ*M, Abcam) and DL-AP5 (100 *μ*M, Abcam), AMPA receptor and NMDA receptor antagonists respectively, were applied in order to block excitatory synaptic activity. Data were recorded using widefield two-photon excitation (*λ* = 780 nm, *P*_*av*_ = 180 mW and an image acquisition rate of 10 Hz) before NBQX/DL-AP5 was added to the bath. The excitation source was then blocked to prevent irradiation of the specimen for 10 minutes, and then further data were recorded using the same excitation and detection parameters.

## Results

**Fig 2:**
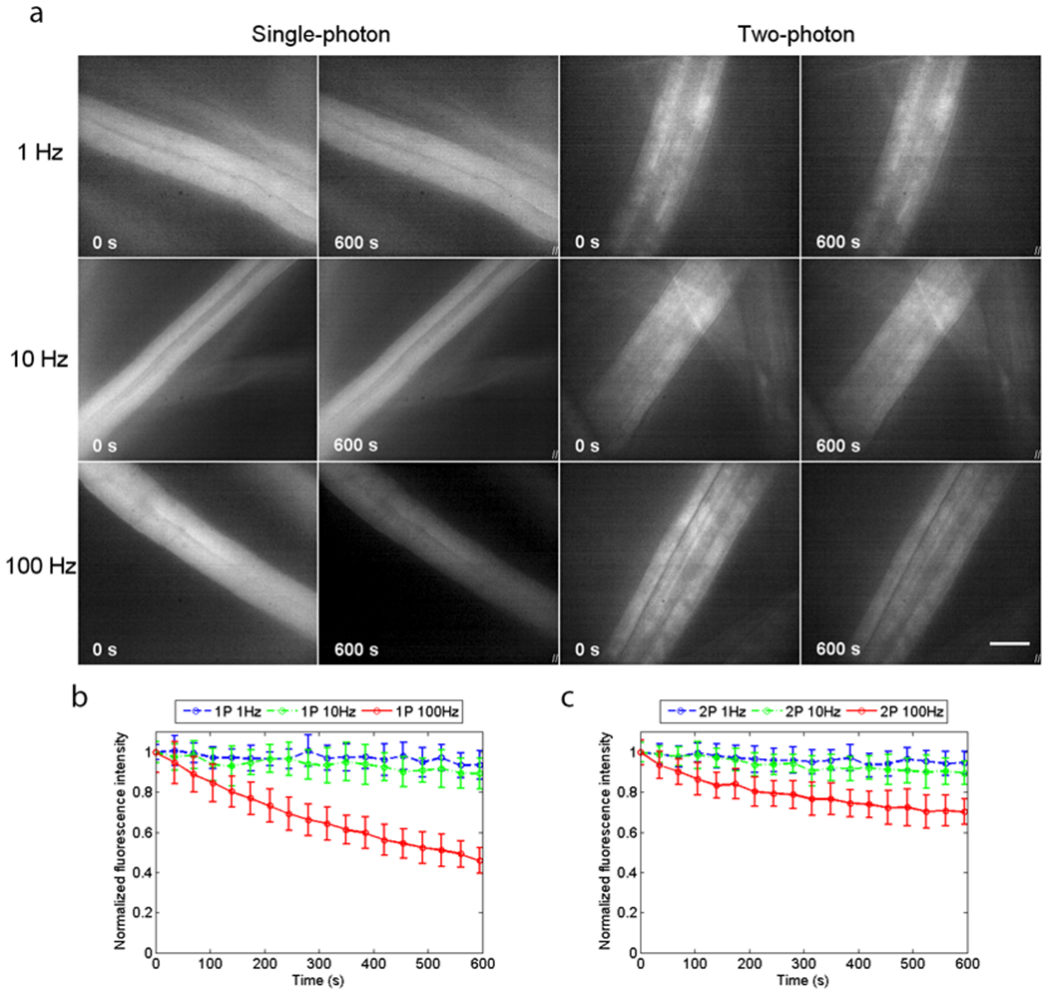
Photo-bleaching experiments using acridine orange. (*a*) Single-photon and two-photon excited widefield images of lens tissue fibres stained with 120 *μ*M acridine orange in PBS, taken at image acquisition rates of 1 Hz, 10 Hz and 100 Hz with continuous irradiation for 600 seconds. The normalised fluorescence intensities, averaged over 30 ROIs from 10 recordings made using 5 specimens for each image acquisition rate are plotted over time in (*b*) for single-photon excitation and in (*c*) for two-photon excitation. Photo-bleaching rates were similar for single-photon and two-photon excitation at image acquisition rates of 1 Hz and 10 Hz, but with an image acquisition rate of 100 Hz, photo-bleaching is markedly reduced with widefield two-photon excitation, with the normalised average fluorescence intensity having levelled off at 75% the initial value, while with single-photon excitation the fluorescence was at 45% after 600 seconds, and still decreasing. Scale bar = 15 *μ*m.

We obtained widefield two-photon excited fluorescence images of lens tissue fibres stained with 120 *μ*M acridine orange in PBS. The specimens were prepared and imaged as described in Materials and Methods. Fig 2a shows the widefield single-photon and widefield two-photon excited fluorescence images of the fibres with continuous irradiation for 600 seconds. The image acquisition rates were 1 Hz, 10 Hz and 100 Hz, and for each imaging condition five specimens were used, with two recordings made for each specimen and the normalised fluorescence intensity measured and averaged over three ROIs within a fibre for each recording. The plots of normalised average fluorescence intensity over time are shown in Fig 2b for single-photon excitation and in Fig 2c for two-photon excitation. With an image acquisition rate of 1 Hz, the plots were similar for single-photon and two-photon excitation, with the normalised average fluorescence intensity having decreased by 5% for two-photon excitation and by 6% for single-photon excitation. The same is true with an image acquisition rate of 10 Hz, the normalised average fluorescence intensity having decreased by 10% for two-photon excitation and by 11% for single-photon excitation. However, with an image acquisition rate of 100 Hz, photo-bleaching is markedly reduced with widefield two-photon excitation, where the normalised average fluorescence intensity had leveled off at 75% of the initial value, while with single-photon excitation the fluorescence was at 45% and still decreasing.

**Fig 3:**
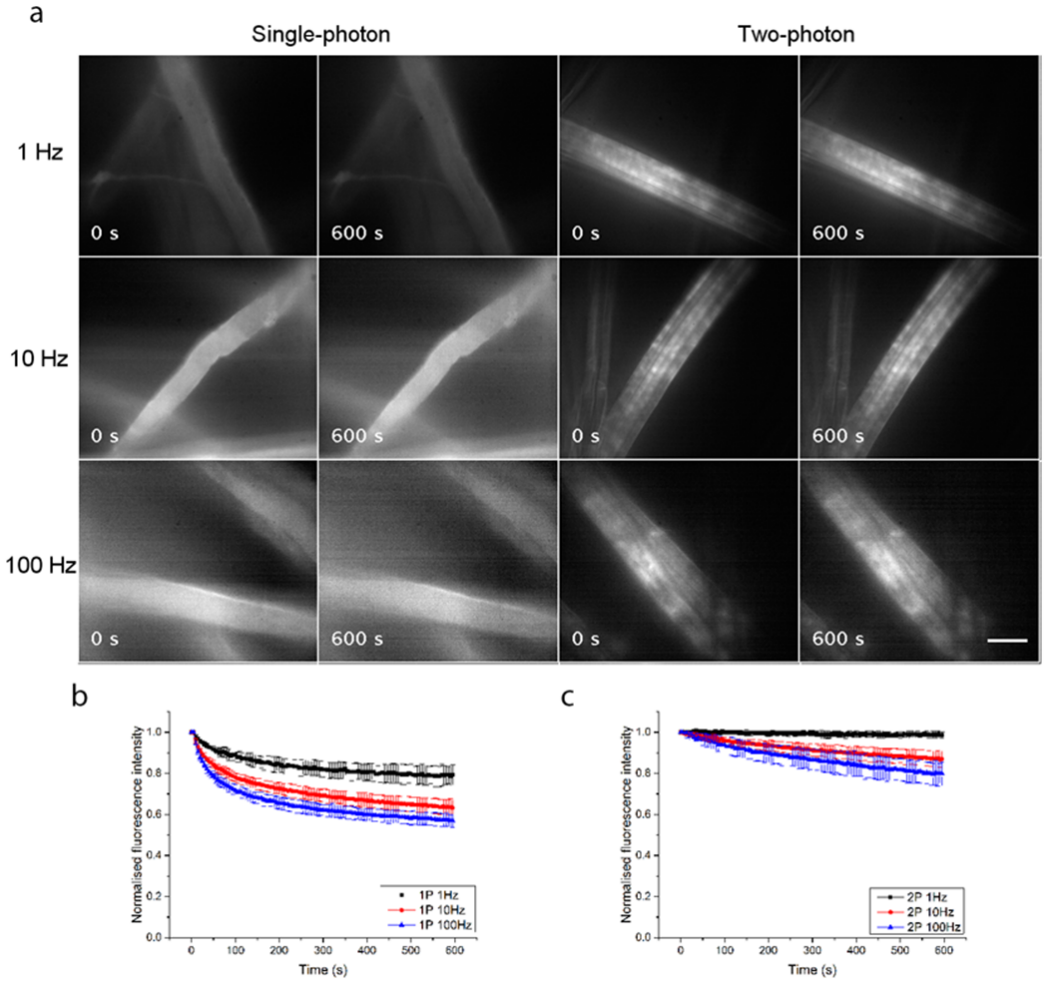
Photo-bleaching experiments using primuline. (*a*) Single-photon and two-photon-excited widefield images of lens tissue fibres stained with 10 *μ*M primuline in tap water, taken at image acquisition rates of 1 Hz, 10 Hz and 100 Hz with continuous irradiation for 600 seconds. The normalised fluorescence intensities, averaged over 18 ROIs from 6 recordings made using 3 specimens for each image acquisition rate are plotted over time in (*b*) for single-photon excitation and in (*c*) for two-photon excitation. Photo-bleaching was consistently reduced with widefield two-photon excitation at all image acquisition rates. Scale bar = 15 *μ*m.

Our experimental results from lens tissue fibres stained with 10 *μ*M primuline in tap water are shown in Fig 3. Fig 3a shows the widefield single-photon and widefield two-photon-excited fluorescence images of the fibres with continuous irradiation for 600 seconds. The image acquisition rates were 1 Hz, 10 Hz and 100 Hz, and for each imaging condition three specimens were used, with two recordings made for each specimen and the normalised fluorescence intensity measured and averaged over three ROIs within a fibre for each recording. The plots of normalised average fluorescence intensity over time are shown in Fig 3b for single-photon excitation and in Fig 3c for two-photon excitation. Photo-bleaching was consistently reduced with widefield two-photon excitation at all image acquisition rates. With an acquisition rate of 1 Hz, the normalised average fluorescence intensity decreased by 21% for single-photon excitation, and only by 1% for two-photon excitation. With an acquisition rate of 10 Hz, it decreased by 37% for single-photon excitation and by 13% for two-photon excitation, and with an acquisition rate of 100 Hz, it decreased by 43% for single-photon excitation and by 20% for two-photon excitation.

Experiments were performed to examine the widefield single-photon and wide-field two-photon-excited fluorescence of FITC Phalloidin-stained 3T3 cells with continuous irradiation for 600 seconds (S1a Fig). The image acquisition rates were 1 Hz, 10 Hz and 100 Hz, and for each imaging condition three specimens were used, with two recordings made for each specimen and the normalised fluorescence intensity measured and averaged over six ROIs. The plots of normalised average fluorescence intensity over time are shown in S1b Fig for single-photon excitation and in S1c Fig for two-photon excitation. We found that as with the photo-bleaching of primuline-stained fibres, the photo-bleaching in fixed cells was consistently reduced with widefield two-photon excitation at all image acquisition rates. With an acquisition rate of 1 Hz, the normalised average fluorescence intensity decreased by 21% for single-photon excitation and only by 12% for two-photon excitation. With an acquisition rate of 10 Hz, it decreased by 50% for single-photon excitation and by 33% for two-photon excitation, and with an acquisition rate of 100 Hz, it decreased by 72% for single-photon excitation and by 55% for two-photon excitation.

**Fig 4:**
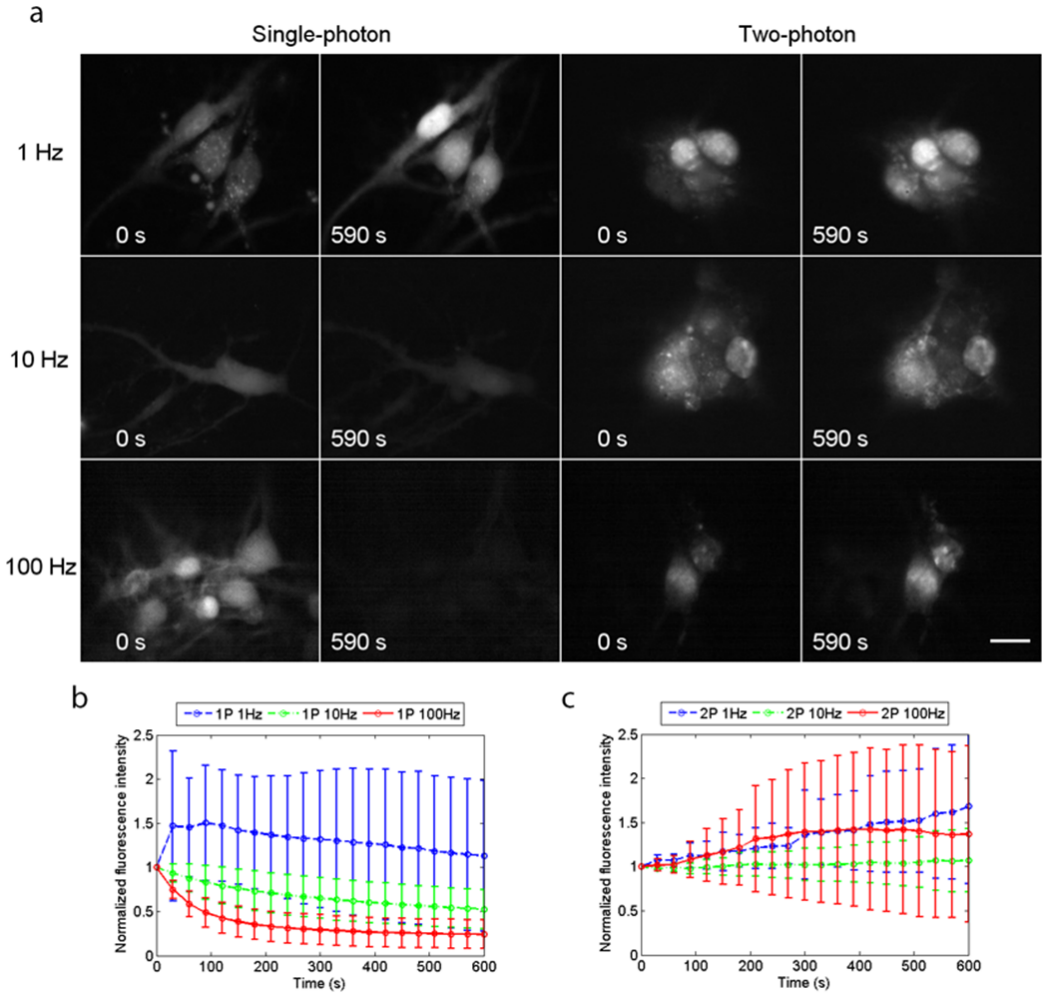
Reduced photo-bleaching using widefield two-photon excitation. (*a*) Single-photon and two-photon excited widefield images of live neuronal cells loaded with Fluo-4 AM, taken at frame rates of 1 Hz, 10 Hz and 100 Hz with continuous irradiation for 590 seconds. The normalised average fluorescence intensities are plotted versus time in (*b*) for single-photon excitation and in (*c*) for two-photon excitation. Photo-bleaching was observed when using single-photon excitation with image acquisition rates of 10 Hz and 100 Hz because of the higher light doses required to compensate for the short exposure times, whereas no photo-bleaching was observed when using two-photon excitation with an acquisition rate of 10 Hz, and there was weak photobleaching in fewer than half of the cell specimens imaged at a rate of 100 Hz, with the main source of error being time-averaged fluctuations in fluorescence signal intensity arising from Ca^2+^ signalling events. Scale bar = 15 *μ*m.

We also obtained high-resolution, high-contrast two-photon excited fluorescence images of Ca^2+^ transients in live neuronal cell bodies at image acquisition rates of up to 100 Hz. Fig 4a shows widefield single-photon and widefield two-photon excited fluorescence images of live cells with continuous irradiation. The image acquisition rates were 1 Hz, 10 Hz and 100 Hz. As described in Materials and Methods, the excitation power was adjusted to give a similar subjective appearance in the first images of all the series. This strategy was adopted to achieve similar signal-to-noise values at the start of all the series. The fluorescence signal intensities for three ROIs located over neuronal cell bodies in each culture dish were measured from time t = 0 to 590 seconds at 5-second intervals using ImageJ [18]. An average value of fluorescence intensity from the three ROIs was obtained for each dataset, and was then normalised relative to the fluorescence signal intensity at time t = 0 seconds. The resultant plots of normalised average fluorescence intensities with time for single-photon excitation and two-photon excitation, averaged over the sample sizes given in Materials and Methods and with standard deviation, are presented as Fig 4b and 4c.

For single-photon and two-photon excitation with an image acquisition rate of 1 Hz, using average powers of ∼50 *μ*W and ∼100 mW respectively, the normalised average fluorescence intensity increased by 13% for single-photon excitation and by 68% for two-photon excitation. With an image acquisition rate of 10 Hz, using average powers of ∼400 *μ*W for single-photon excitation and ∼170 mW for two-photon excitation, the normalised average fluorescence intensity decreased by 47% for single-photon excitation whereas that for two-photon excitation increased by 7%. With an image acquisition rate of 100 Hz, using average powers of ∼2.5 mW for single-photon excitation and 360 mW for two-photon excitation, there was a rapid decrease in the normalised average fluorescence intensity for single-photon excitation, with almost no fluorescence recorded after 590 seconds, whereas it increased by 37% for two-photon excitation. The increase in the average fluorescence intensity over time is a combination of an increase in baseline fluorescence intensity and time-averaged fluctuations in fluorescence intensity arising from Ca^2+^ signalling events. So even at fast image acquisition rates (with increased irradiation to compensate for the shorter integration time), useful images were being obtained with widefield two-photon excitation long after the conventional single-photon recordings had faded totally.

An analysis of the signal-to-noise ratios in images of Ca^2+^ transients in live neuronal cell bodies is not trivial because of the complication of fluctuations in fluorescence signal intensity arising from Ca^2+^ signalling events, but we have made measurements of both the signal-to-noise ratio of the fluorescence signal for single-photon and two-photon datasets obtained at the different imaging speeds. We achieved this by eliminating any Ca^2+^ spikes from our analysis (we define these conditionally as *(a)* having the definitive shape of a Ca^2+^ spike and *(b)* having a change in normalised fluorescence intensity of +0.1 or greater) and considering only the fluorescence baseline. We have also made measurements of the noise in the background signal, which is not complicated by cell signalling events. These data are presented in S1 Table. We chose an initial fluorescence signal intensity of between 1,000–2,000 counts for low-light imaging which was far from saturation (65,536 counts for our 16-bit imaging detector) to avoid unnecessary photo-bleaching at all image acquisition rates. The average single-photon and two-photon excited fluorescence signal intensity counts are similar (to within a factor of two), and the standard deviation across this range is within 15% of the average signal, with the greatest standard deviation measured for the single-photon datasets at the 1 Hz and 10 Hz imaging speeds. In all datasets analysed, the standard deviation may include small changes in Ca^2+^ that we do not eliminate as spikes, which increases the measured standard deviations. The signal to background ratio for each dataset is also similar, to within a factor of approximately two across the range of imaging speeds for both single-photon and two-photon excitation.

S1 Video shows time-lapse recordings of live neuronal cell bodies loaded with Fluo-4 AM at a frame rate of 100 Hz using single-photon (left) and two-photon (right) widefield excitation. The neuronal cultures were irradiated continuously for 590 seconds and the video shows a frame every 5 seconds, demonstrating very clearly the rapid decrease in fluorescence intensity observed using widefield single-photon excitation, while no significant reduction in fluorescence intensity was observed using widefield two-photon excitation.

**Fig 5:**
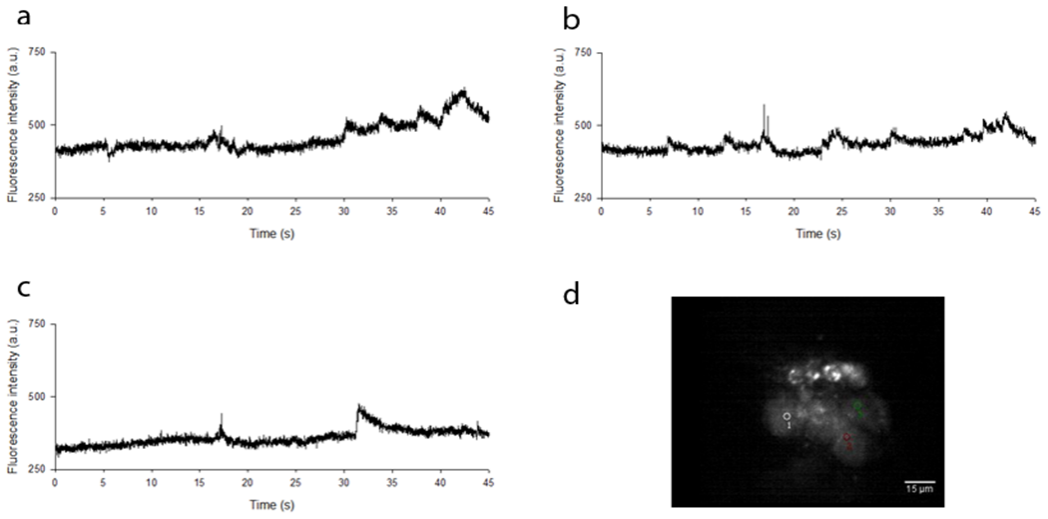
Localisation of changes in fluorescence intensity. *(a)*, *(b)* and *(c)* show widefield two-photon excited fluorescence intensities within three adjacent live neuronal cell bodies loaded with Fluo-4 AM, acquired at a frame rate of 100 Hz over a duration of 45 seconds. This confirms that the measured change in fluorescence signal intensity with time was not global across the image, but was instead localised to individual cell bodies at different times. *(d)* An image of the field of view and accompanying ROIs from which the measurements were taken.

To confirm the two-photon nature of the excitation process, we obtained images of live neurones at image acquisition rates of 1 Hz, 10 Hz, 50 Hz and 100 Hz at five time-averaged incident powers. Log-log plots of fluorescence intensity from five ROIs with excitation power gave gradients of between 1.8 and 2.3 for all frame rates, confirming two-photon excitation. An example time course with three ROIs is shown in Fig 5, and S2 Video shows slow (∼seconds) Ca^2+^ transients at an image acquisition rate of 100 Hz over a duration of 10 seconds. For display purposes, the video file has been reduced in size by 50%. In this video, three ROIs were chosen in three adjacent cell bodies to not only demonstrate the rate of image capture but also to confirm that the measured change in fluorescence signal intensity with time was not global across the image, but is localised to individual cell bodies at different times. This evidence suggested that the change in measured fluorescence signal intensity was not a consequence of fluctuations in laser power or camera instability, but arose from localised changes in intracellular Ca^2+^ concentration.

**Fig 6:**
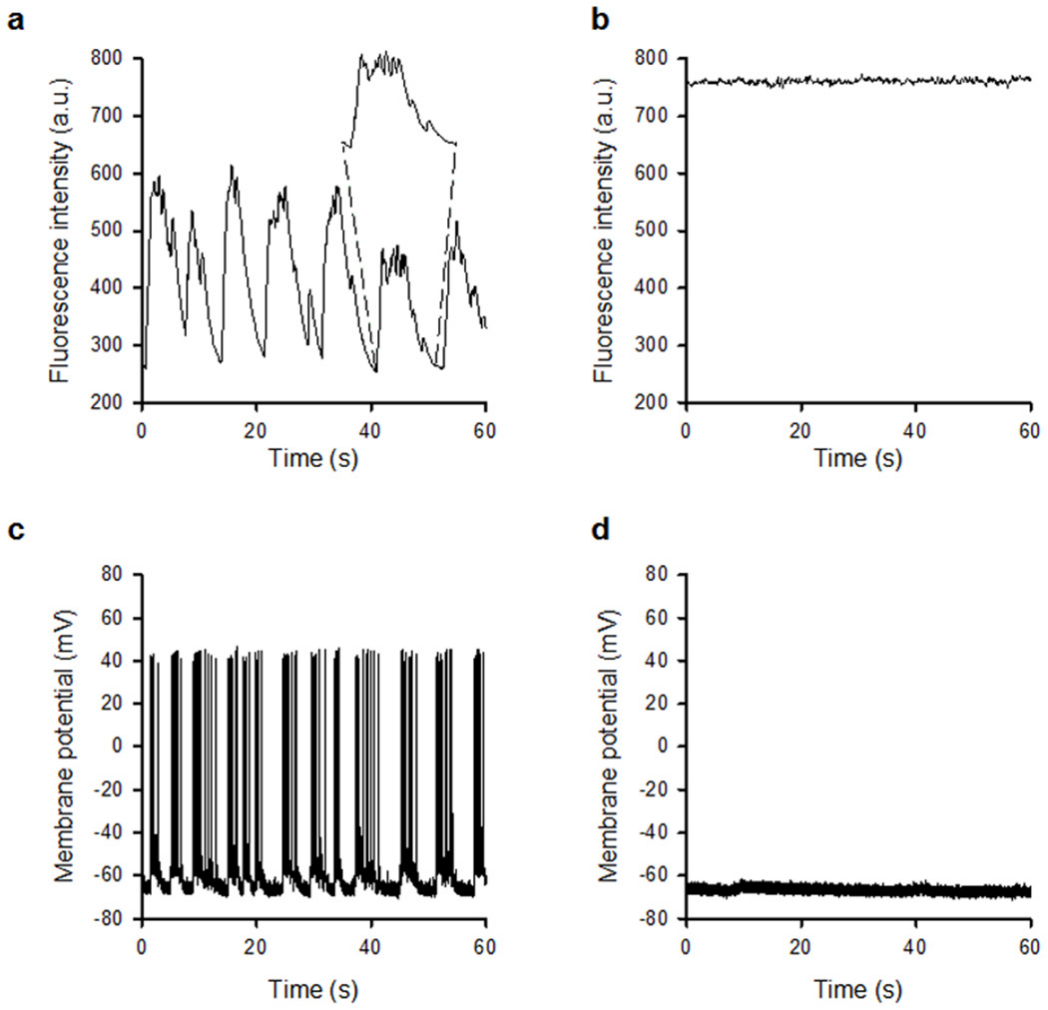
Widefield two-photon excitation for recording synaptic activity. (*a*) Fluorescence recording at a frame rate of 10 Hz shows spontaneous changes in fluorescence intensity in live neuronal cell bodies loaded with Fluo-4 AM, which were abolished in the presence of the glutamatergic antagonists DL-AP5 and NBQX (*b*). The elevated Ca^2+^ level seen in Fig 5b fits with the observation of an overall increase in two-photon excited fluorescence signal over time at an image acquisition rate of 100 Hz shown in Fig 4. The expanded region of (*a*) shows the envelope of events indicating synaptically-driven activity. (*c*) Whole-cell current clamp recordings from hippocampal neurones revealing spontaneous action potential firing which is abolished in the presence of DL-AP5 and NBQX (*d*).

Having established that widefield two-photon excitation reduces photo-bleaching at high frame rates, we then examined whether Ca^2+^ events induced by synaptic activity could be observed in primary hippocampal cultures. Fig 6a and S3 Video (left panel), acquired at a frame rate of 10 Hz, show spontaneous changes in fluorescence intensity in live neuronal cell bodies over a 60-second time period, which were abolished in the presence of the AMPA and NMDA receptor antagonists NBQX (20 *μ*M) and DL-AP5 (100 *μ*M) (Fig 6b and right panel of S3 Video), indicating that these events are driven by glutamatergic excitatory synaptic activity. The elevated Ca^2+^ level seen in Fig 6b fits with the observation of an overall increase in two-photon excited fluorescence signal over time shown in Fig 4. The sensitivity to NBQX/DL-AP5 is similar to that observed when using the whole-cell patch clamp technique to monitor synaptically driven events including spontaneous action potential firing using identical cultures (Fig 6c and 6d) [17, 19] thus highlighting the functional capability of this method.

Since the cell preparations used here were a thin monolayer of cells, we did not perform optical sectioning. However, weak optical sectioning of thicker specimens was possible. S2 Fig shows optical sectioning of an auto-fluorescent fixed *Taraxacum* pollen specimen mounted in Histomount, obtained at an excitation wavelength of *λ* = 820 nm and using the same chromatic reflectors and emission filters shown in Fig 1. Here a 60x/1.35 NA oil immersion objective was used, and the montage was obtained by moving the specimen by 1 *μ*m increments axially over a range of 25 *μ*m. The frame rate was set to 10 Hz for each image. The spikes at the top and bottom of the pollen grain are clearly independently resolved without deconvolution or other image processing methods. The optical sectioning shown in S2 Fig is closely similar to that in a widefield single-photon fluorescence microscope, confirming that the optical depth of field is due to the focusing of the emission only.

**Fig 7:**
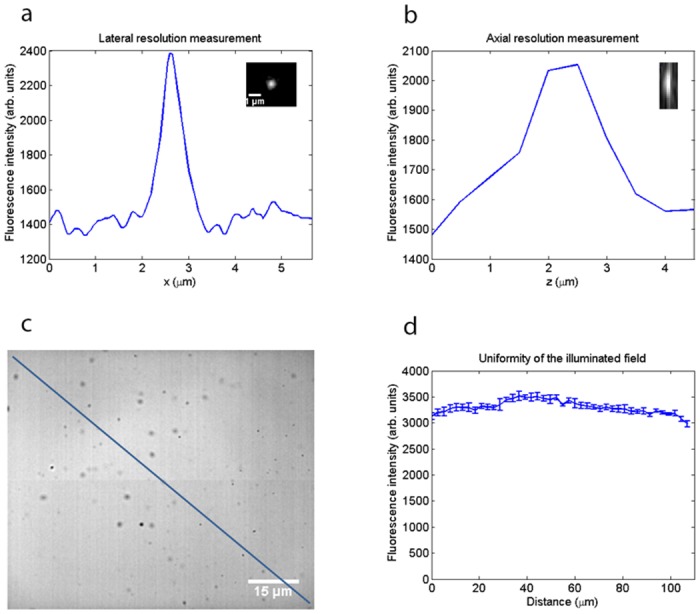
Resolution measurements and evaluation of the uniformity of the illuminated field. *(a)* Lateral intensity profile through a 200 nm bead imaged using a 60x/1.35 NA oil immersion lens at an excitation wavelength of 820 nm, showing the lateral resolution to be 0.55 *μ*m from the full width at half maximum. *(b)* Axial intensity profile of the same bead, showing that the axial resolution is 1.5 *μ*m. *(c)* Widefield two-photon image of a fluorescent Perspex block used to evaluate the uniformity of the illuminated field. Scale bar = 15 *μ*m. *(d)* Intensity profile along the diagonal line in *(c)*, showing the fluorescence intensity varied by less than 10% across the field of view.

By imaging 200 nm fluorescent beads (Fluoresbrite YG microspheres, Polysciences) using a 60x/1.35 NA oil immersion lens (n = 1.5) at a wavelength of 820 nm, we measured a lateral resolution of 0.55 *μ*m and an axial resolution of 1.5 *μ*m from the full width at half maximum (FWHM) of the lateral and axial intensity profiles, respectively, shown in Fig 7a and 7b. These resolution values are not as good as the optical resolution of a two-photon microscope with a diffraction-limited focus, given by the FWHM of the lateral and axial intensity-squared profiles [20]:

$$\begin{array}{c} FWHM_{xy}=\frac{0.325\sqrt{2\;ln2}\;\lambda }{NA^{0.91}}=\frac{0.325\sqrt{2\;ln2}*0.82\;\mu m}{1.35^{0.91}}=0.24\;\mu m \end{array}$$ (5)

and

$$\begin{array}{c} FWHM_{z}=\frac{0.532\sqrt{2\;ln2}\;\lambda }{n-\sqrt{n^{2}-NA^{2}}}=\frac{0.532\sqrt{2\;ln2}*0.82\;\mu m}{1.5-\sqrt{1.5^{2}-1.35^{2}}}=0.61\;\mu m, \end{array}$$ (6)

but this is an expected consequence of weak focusing of the excitation beam. To confirm that the uniformity of the field was acceptable for imaging, we performed widefield two-photon excitation of a fluorescent Perspex block (Chroma). Fig 7c shows a two-photon excited fluorescence image of this specimen obtained using an image capture rate of 10 Hz, with a diagonal line indicating the region of interest from which the intensity profile (Fig 7d) was taken using ImageJ. The error bars show the standard deviation from an average of four images. It is clear from the intensity profile that the illuminated field was not perfectly homogeneous, but the fluorescence intensity level varied by less than 10% across the field of view.

## Discussion

In evaluating this widefield two-photon microscopy method, it is natural to ask what advantages it confers in relation to single-photon widefield microscopy.

The most important advantage of the method is the low photo-bleaching rate compared with widefield single-photon excitation when applied to the imaging of fast events. This would appear to present a great experimental advantage in applications such as calcium imaging, as problems with photo-bleaching when using Ca^2+^ indicators are well-known [1], severely limiting the number of experiments possible with a given specimen and restricting the useful duration of imaging experiments.

It is accepted that the main reason for reduced photo-bleaching in two-photon point-scanning microscopy is the confined nature of the excitation source [21]. In our widefield two-photon microscope, we instead use a weakly focused beam with a much larger incident spot and longer axial response, with an illumination pattern more closely resembling a single-photon epi-fluorescence microscope, albeit with an infrared Ti:Sapphire laser instead of an incoherent visible source. The overall volume of excitation is increased using our method in comparison to point-scanning two-photon microscopy, but we are also applying a much reduced peak intensity excitation. This is important because the higher photon flux used in standard point-scanning two-photon microscopy is known to lead to the devastating higher-order photon interactions that induce photo-bleaching [3]. Our lower intensity excitation, spread over a large volume, is likely responsible for the low photo-bleaching that we observe in our imaging experiments. Additionally, in two-photon widefield mode the photometric volume is much larger than the point spread function of the objective, and the infrared intensity is vastly lower, so it is very unlikely that the fluorophore is ever saturated. This makes transitions to the triplet state, which is understood to have a significant role in photo-bleaching, less likely [22]. This may be the main reason for the lower rate of photo-bleaching in two-photon widefield microscopy.

Although recent approaches to point-scanning two-photon excitation such as the LOTOS-based method of Konnerth and co-workers has increased the image acquisition rate dramatically, allowing even faster imaging with reduced photo-bleaching and phototoxicity [9–11], the LOTOS method gives a reduced field of view, and it is technically challenging and complex and comes at a high cost. Widefield two-photon microscopy is easy to implement, with no costly additions to the microscope, and we suggest that this method may prove advantageous when using fluorophores or photoproteins with fast photo-bleaching rates, including the Ca^2+^-sensitive dyes, CFP [23], eBFP [24] and YFP [25]. This technique may also reduce unwanted photo-bleaching in FRAP, FLAP and other similar experimental methods, where photo-bleaching of the untargeted region can compromise results [26, 27]. We have used a single camera and fluorophore here, but our method could be easily adapted for simultaneous multi-channel recording using an image splitter and a second sCMOS camera.

The mechanisms responsible for light-induced increases in fluorescence baseline signal intensity over time are not well understood. Increased fluorescence has often been observed and it has been suggested that this results from the photo-induced release of Ca^2+^ [28, 29] or from direct photo-activation of the fluorescent dye [30, 31] or effects on membrane permeability of sample heating [32], all of which can be expected at the short wavelengths applied in single-photon microscopy. The more common problem of photo-bleaching due to the destruction of the dye by free-radicals generated by illumination [33] is a serious limitation in all single-photon live cell imaging. Our present results suggest that widefield two-photon microscopy may overcome the bleaching problem when using image acquisition rates between 10–100 Hz.

The benefit in time-resolution is plainly shown by the widefield two-photon results at high image acquisition rates. The penetration ability of two-photon excitation, as compared with single-photon excitation, which has been well established [34], has not been measured here, but may be expected in this method also, because the low absorption of the infrared excitation wavelengths is still retained in widefield two-photon excitation although the lower scattering of the longer excitation wavelengths is less important for a distributed excitation spot: the already large excitation volume is not substantially increased by the scattering. However, the widefield two-photon method cannot be expected to have an optical sectioning capability as good as that in conventional scanned two-photon excitation. By imaging 200 nm fluorescent beads using a 60x/1.35 NA oil immersion lens at *λ* = 820 nm, we measured an axial resolution of 1.5 *μ*m and a lateral resolution of 0.55 *μ*m. These resolution values are not as good as those for a point-scanning two-photon microscope with similar excitation parameters (0.61 *μ*m and 0.24 *μ*m, respectively), but they are an expected consequence of weak focusing of the excitation beam. These results convincingly show the benefit of widefield two-photon microscopy for a cell monolayer, but at present, because of the reduced optical sectioning we expect that the system would be less suitable for thick specimens, including whole-animal preparations. However, it is conceivable that further optical development may support improved optical sectioning for three-dimensional imaging. For example, although no temporal focusing [35] was used here, our widefield method could be used with temporal focusing to obtain improved optical sectioning, without the low image contrast and much reduced resolution observed in multi-focal two-photon microscopy [36].

Ultra-short pulsed near-infrared lasers of the type used here have been previously used to generate Ca^2+^ waves in differentiated cells. Smith *et al* [37] used a single diffraction-limited beam focus of *w*_0_ = 0.3 *μ*m, with an average power *P*_*av*_ > 20 mW, *λ* = 775 nm, repetition frequency Δ*ν* = 82 MHz and pulse duration *τ* = 140 fs, which gives a peak intensity *I*_*peak*_ > 6.16 × 10^15^ W/m^2^. However, since the peak intensity of illumination used in our two-photon widefield microscope is around three orders of magnitude less than Smith *et al* used in their experiments, it is highly unlikely that we are observing light-induced Ca^2+^ transients. Indeed, the sensitivity of the observed Ca^2+^ events to blockade of glutamatergic synaptic activity indicates that the events are synaptically driven and are presumably triggered by action potentials arriving at the neuronal cell body, the consequence of which is the transient increase in intracellular Ca^2+^ level due to membrane potential depolarisation. The close similarity between the optical transients observed here and those recorded electrically in individual cells by the whole-cell path clamp technique in current clamp mode are a clear demonstration of the sensitivity, time resolution and usefulness of this method. We have concentrated here on the improved time resolution and remarkably low level of photo-bleaching, but, as already pointed out by Hwang *et al*, there may be other advantages over single-photon imaging, such as improved discrimination against auto-fluorescence and clearer imaging of dense tissues. We believe that the new method may have particular value with biological specimens, not only neuronal, where the longevity of the preparation under imaging conditions is more important than the precision of optical sectioning. This may include photoprotein-labelled cells within functioning tissues.

## Supporting Information

S1 Table Signal-to-noise ratio in images of Ca^2+^ transients in live neuronal cell bodies. We chose an initial fluorescence signal intensity of between 1,000–2,000 counts for low-light imaging which was far from saturation (65,536 counts for our 16-bit imaging detector) to avoid unnecessary photo-bleaching at all image acquisition rates. The average single-photon and two-photon excited fluorescence signal intensity counts are similar (to within a factor of two), and the standard deviation across this range is within 15% of the average signal, with the greatest standard deviation measured for the single-photon datasets at the 1 Hz and 10 Hz imaging speeds. The signal to background ratio for each dataset is also similar, to within a factor of approximately two across the range of imaging speeds for both single-photon and two-photon excitation.
(DOC)

S1 Fig Photo-bleaching experiments using fixed cells. (*a*) Single-photon and two-photon-excited widefield images of 3T3 cells stained with FITC Phalloidin, taken at image acquisition rates of 1 Hz, 10 Hz and 100 Hz with continuous irradiation for 600 seconds. The normalised fluorescence intensities, averaged over 36 ROIs from 6 recordings made using 3 specimens for each image acquisition rate are plotted over time in (*b*) for single-photon excitation and in (*c*) for two-photon excitation. Photo-bleaching in the cellular specimens was consistently reduced with widefield two-photon excitation at all image acquisition rates. Scale bar = 15 *μ*m.
(TIF)

S2 Fig Optical sectioning of pollen specimen. Widefield two-photon microscopy shows weak optical sectioning of an auto-fluorescent fixed *Taraxacum* pollen specimen, obtained by moving the specimen by 1 *μ*m increments axially over a range of 25 *μ*m. No post-processing was performed on the images except for cropping to display only a single pollen grain within the image field. The optical sectioning shown here is closely similar to that obtained in a widefield single-photon fluorescence microscope. This confirms that the optical depth of field is due to the focusing of the emission only. Scale bar = 15 *μ*m.
(TIFF)

S1 Video Reduced photo-bleaching using widefield two-photon excitation. Time-lapse recordings of live neuronal cells loaded with Fluo-4 AM at a frame rate of 100 Hz using single-photon (left) and two-photon (right) widefield excitation. The neuronal cultures were irradiated continuously for 590 seconds and the video shows a frame every 5 seconds, demonstrating very clearly the rapid decrease in fluorescence intensity observed using widefield single-photon excitation, while no significant reduction in fluorescence intensity was observed using widefield two-photon excitation. Scale bar = 15 *μ*m.
(MOV)

S2 Video Localisation of changes in fluorescence intensity. Widefield two-photon excitation microscopy shows Ca^2+^ transients in live neuronal cell bodies loaded with Fluo-4 AM at a frame rate of 100 Hz over a duration of 10 seconds. Three regions of interest (white, red and green) are shown in three adjacent cell bodies to demonstrate not only the rate of image capture but to confirm that the measured change in fluorescence signal intensity with time was not a consequence of fluctuations in laser power or camera instability, but arose from localised changes in intracellular Ca^2+^ concentration. Scale bar = 15 *μ*m.
(MOV)

S3 Video Confirmation that the Ca^2+^ events are synaptically driven. (*A*) Spontaneous Ca^2+^ events recorded in live neuronal cells over a 60-second time period before the application of the glutamatergic antagonists NBQX and DL-AP5. (*B*) Video recorded from the same region after the application of NBQX and DL-AP5, showing no Ca^2+^ events. Scale bar = 15 *μ*m.
(MOV)
