# Supplementary material for: Widefield Two-Photon Excitation without Scanning: Live Cell Microscopy with High Time Resolution and Low Photo-Bleaching
Source: PLoS One. 2016 Jan 29;11(1):e0147115. doi: 10.1371/journal.pone.0147115 (PMC4732674; doi:10.1371/journal.pone.0147115)
Supplement: S1 Table — We chose an initial fluorescence signal intensity of between 1,000–2,000 counts for low-light imaging which was far from saturation (65,536 counts for our 16-bit imaging detector) to avoid unnecessary photo-bleaching at all image acquisition rates. The average single-photon and two-photon excited fluorescence signal intensity counts are similar (to within a factor of two), and the standard deviation across this range is within 15% of the average signal, with the greatest standard deviation measured for the single-photon datasets at the 1 Hz and 10 Hz imaging speeds. The signal to background ratio for each dataset is also similar, to within a factor of approximately two across the range of imaging speeds for both single-photon and two-photon excitation. (DOC) [file pone.0147115.s001.doc]

| Frame rate | Single-photon excitation |  |
| --- | --- | --- |
|  | Fluorescence signal intensity (counts) | Background signal intensity (counts) |
| 1 Hz (n=11) | 1222.20 ± 177.02 | 110.38 ± 1.41 |
| 10 Hz (n=20) | 1621.87 ± 242.90 | 231.98 ± 2.37 |
| 100 Hz (n=10) | 1326.94 ± 154.47 | 95.00 ± 1.15 |
|  |  |  |
|  | Two-photon excitation |  |
|  | Fluorescence signal intensity (counts) | Background signal intensity (counts) |
| 1 Hz (n=26) | 2003.29 ± 258.18 | 291.60 ± 3.31 |
| 10 Hz (n=30) | 1174.63 ± 127.81 | 122.39 ± 4.46 |
| 100 Hz (n=10) | 1246.54 ± 86.32 | 198.62 ± 4.81 |
