# Supplementary figures and images for: Widefield Two-Photon Excitation without Scanning: Live Cell Microscopy with High Time Resolution and Low Photo-Bleaching
Source: PLoS One. 2016 Jan 29;11(1):e0147115. doi: 10.1371/journal.pone.0147115 (PMC4732674; doi:10.1371/journal.pone.0147115)

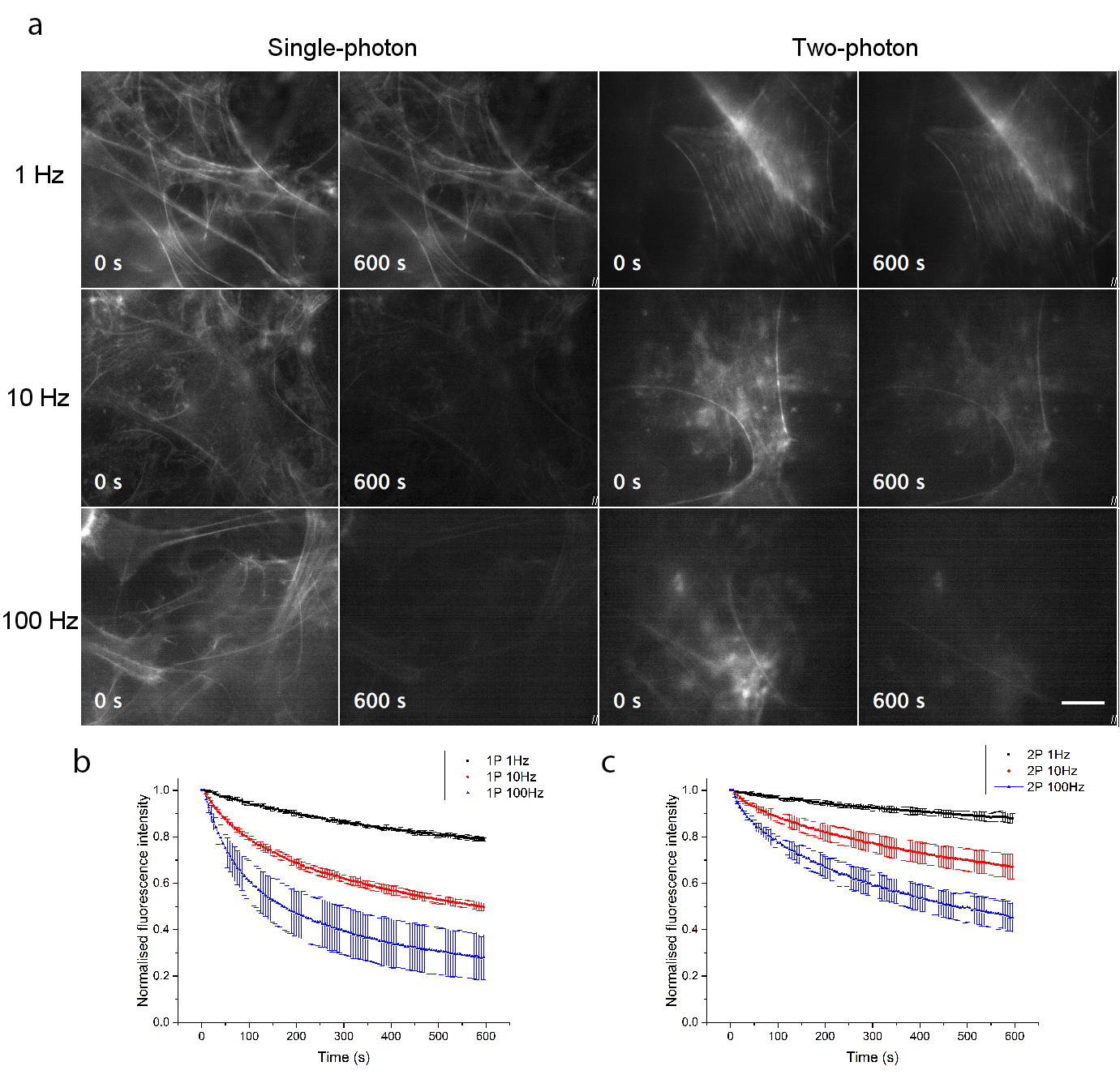

Supplement: S1 Fig — (a) Single-photon and two-photon-excited widefield images of 3T3 cells stained with FITC Phalloidin, taken at image acquisition rates of 1 Hz, 10 Hz and 100 Hz with continuous irradiation for 600 seconds. The normalised fluorescence intensities, averaged over 36 ROIs from 6 recordings made using 3 specimens for each image acquisition rate are plotted over time in (b) for single-photon excitation and in (c) for two-photon excitation. Photo-bleaching in the cellular specimens was consistently reduced with widefield two-photon excitation at all image acquisition rates. Scale bar = 15 μm. (TIF) [file pone.0147115.s002.tif]

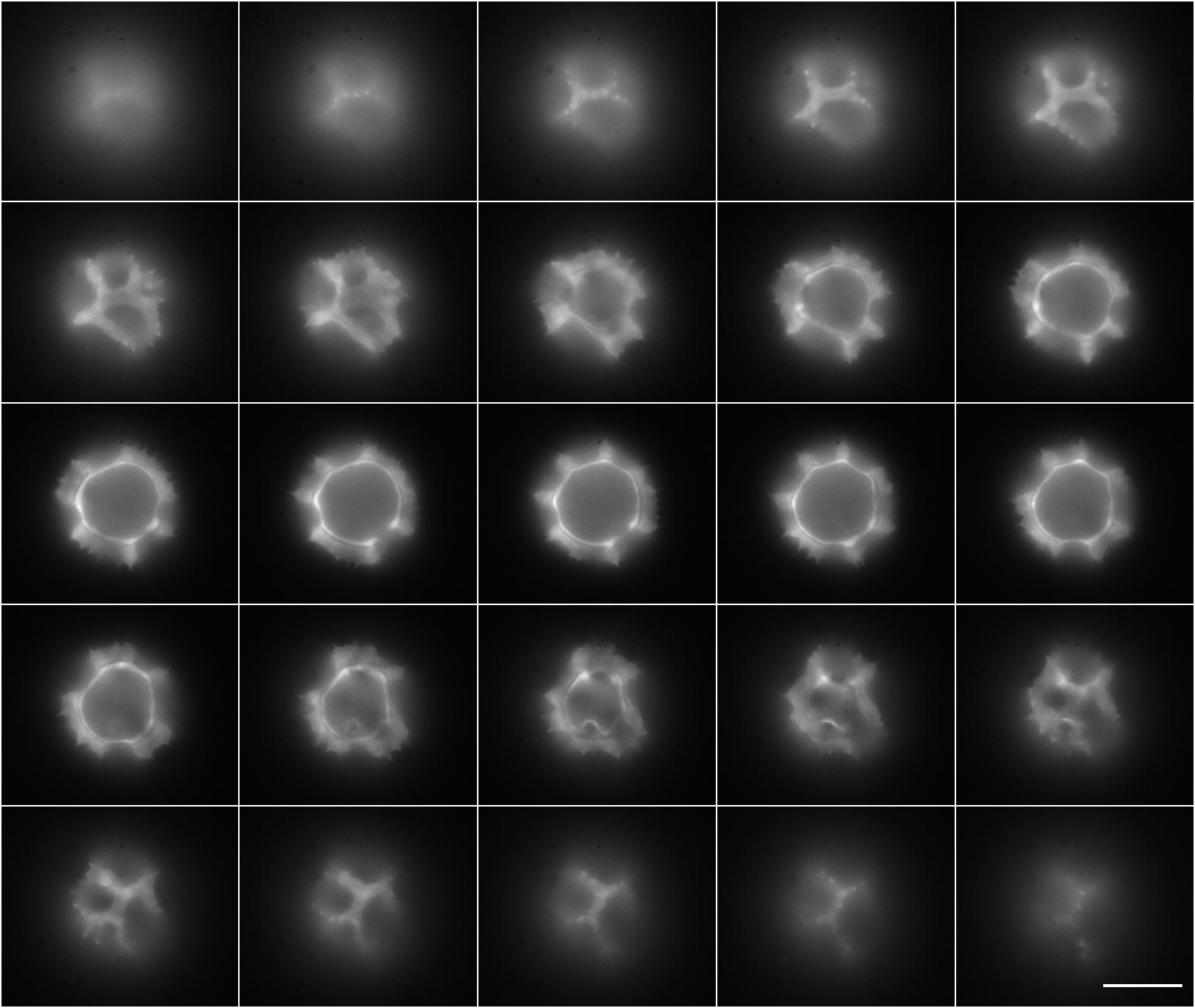

Supplement: S2 Fig — Widefield two-photon microscopy shows weak optical sectioning of an auto-fluorescent fixed Taraxacum pollen specimen, obtained by moving the specimen by 1 μm increments axially over a range of 25 μm. No post-processing was performed on the images except for cropping to display only a single pollen grain within the image field. The optical sectioning shown here is closely similar to that obtained in a widefield single-photon fluorescence microscope. This confirms that the optical depth of field is due to the focusing of the emission only. Scale bar = 15 μm. (TIFF) [file pone.0147115.s003.tiff]
